# Diagnostic yield of gastrointestinal endoscopy in North West Region Cameroon and trends in diagnosis over time

**DOI:** 10.11604/pamj.2018.29.178.10785

**Published:** 2018-03-27

**Authors:** Jacob Kamdem, Dennis Palmer, Charles Barrier, Richard Bardin, James Allen Brown, Mark Topazian

**Affiliations:** 1 Mbingo Baptist Hospital, Bamenda, Cameroon; 2 Carolina Medical Center, University of North Carolina, USA; 3 Mayo Clinic, USA

**Keywords:** Diagnosis, therapy, stomach, colon, endoscopy, colonoscopy, Africa, Cameroon, ulcer, cancer

## Abstract

**Introduction:**

Gastrointestinal endoscopy is an important modality for diagnosis and treatment of gastrointestinal disease, but there are limited data regarding the diagnostic yield of endoscopy in Cameroon and changes in the prevalence of endoscopic findings over time. Our aims were to describe the diagnostic utility of esophagogastroduodenoscopy (EGD) and colonoscopy, the impact of periodic on-site mentorship on cecal intubation rate and changes in the prevalence of common upper gastrointestinal findings when compared to a similar report from our region published in 1990.

**Methods:**

Retrospective review of all EGD and colonoscopy procedures performed during 2015 at a regional referral hospital in North West Region, Cameroon, with comparison to EGD findings reported by Dent and colleagues in 1990. During the year 3 endoscopists provided periodic colonoscopy mentorship.

**Results:**

Among 1,371 EGDs, abnormalities were found in 59.7% and therapeutic interventions (most commonly esophageal stricture dilation or band ligation of varices) were performed in 137 (10%). When compared to 25 years previously, peptic ulcer disease was less common and esophagitis was more common (p < 0.0001; p < 0.0001). The prevalence of malignancy (2.2%) was similar. Among 380 colonoscopies diagnostic findings were seen in 60.5%, including colorectal malignancies in 5.8%. Cecal intubation rate improved from 32% to 89% during the one-year study period.

**Conclusion:**

EGD and colonoscopy have a diagnostic yield of about 60% in symptomatic patients in North West Region, Cameroon. When compared to 1990 peptic ulcer disease was less common and esophagitis was more common. Periodic on-site mentorship was associated with improved physician performance of colonoscopy.

## Introduction

Gastrointestinal endoscopy, including esophagogastroduodenoscopy (EGD) and colonoscopy, is the investigation of choice for many gastrointestinal diseases and is used for both diagnosis and treatment [1]. Endoscopic biopsies are useful for diagnosis of cancer, inflammation and helicobacter pylori infection, a precancerous condition [2-5]. In developing countries many hospitals do not offer endoscopy. In the Northwest (NW) Region of Cameroon, as far as we are aware, endoscopy has only been available in recent years at Mbingo Baptist Hospital (MBH), a regional referral hospital where this study took place. Barriers to a sustainable endoscopy service include the cost to purchase, disinfect and maintain endoscopy equipment and the limited opportunities available for training endoscopists. Previous studies have assessed the utility and diagnostic yield of gastrointestinal endoscopy in sub Saharan Africa. These studies report positive findings in 57% to 81% of EGD procedures and about 60% of colonoscopy procedures [6-8]. A previous study of EGD was reported from Northwest Region of Cameroon in 1990 [6]. The aims of our study were to determine the diagnostic yield of both EGD and colonoscopy in North West Region of Cameroon, to assess changes in colonoscopy performance following periodic mentorship and to compare EGD findings with what was found 25 years ago in this same region.

## Methods

This was a retrospective study of all EGDs and colonoscopies performed at MBH from 02 January, 2015 to 31 December, 2015. We included every patient of both sexes who underwent an EGD or colonoscopy exam. Complete EGD was defined as passage of the endoscope to the second portion of duodenum, or to an obstructing lesion that prevented passage of the endoscope. Similarly, colonoscopy was considered complete when the cecum was examined or when an obstructing lesion was reached. “Cecal intubation rate” was calculated as the rate at which the cecum was reached in patients without an obstructing lesion. We excluded from this study patients who were referred for but did not undergo endoscopy, either because they did not consent or were not cooperative with the examination, preventing passage of the endoscope. Data was abstracted from the endoscopy register, as well as pathology reports and descriptive statistics were performed. This methodology is similar to previous studies from other African endoscopy units [9].

Every patient undergoing endoscopy was seen in outpatient clinics or admitted to hospital wards and endoscopy was ordered by their treating physician or surgeon. The fee for endoscopy was 15,000f cfa, approximately equal to 25 American dollars. The patient was informed about the procedure and a verbal consent obtained prior to endoscopy. Except in children, endoscopy was performed without sedation and the patient had to be nil per os (NPO) for at least 6 hours prior to endoscopy. Topical oro-pharyngeal anesthesia (lidocaine 2%) was provided for EGD. Patients undergoing colonoscopy took a colonic preparation consisting of a clear liquid diet the day before the procedure, 6 Bisacodyl tablets orally at 4 pm the day before the procedure, nothing by mouth after midnight and tap water enemas until clear on the morning of the procedure. Monitoring of vital signs and oxygen saturation was performed in acutely ill patients and all those receiving sedation, and supplemental oxygen (via nasal cannula) was given as needed. All patients undergoing endoscopy during the study period had a written report placed in the endoscopy unit’s register including hospital number, name, age, sex, address, procedure and endoscopy findings, and these were reviewed.

Examinations were performed with an Olympus EXERA CLV-160 endoscopy system and CF TQ160 and GF XQ 140 video colonoscopes and endoscopes. Biopsies were obtained when a suspect lesion was seen. Multiple biopsy pieces where collected in a solution of 10% formaldehyde and analyzed in the hospital’s pathology department. Endoscopes were disinfected after each use following international guidelines, including manual cleaning with a detergent solution and disinfection in 2% Glutaraldehyde. Examinations were performed by Cameroonians who had received previous training, including a senior medical resident (JK) who performed 2/3 of the procedures, as well as other medical and surgical residents. During the course of the year three endoscopists visited the hospital and provided additional instructions in endoscopic technique, particularly colonoscopy. These visits each lasted 2 to 4 weeks, and included daily mentorship of the first author and other residents rotating on the endoscopy service.

Data were analyzed with on-line statistical software, using openEpi-2x2 Table statistics; and a Fisher’s two-tail exact test was used to compare our EGD data with those reported by Dent in 1990 [6]. Dent did not report any colonoscopy data.

## Results

1752 endoscopic procedures were performed during the study period, including 1371 (78.2%) EGDs and 380 (21.7%) flexible sigmoidoscopies and colonoscopies. The mean patient age was 47.6 (range 2 to 91 years); 56.5% were younger than 50 years of age and 53.1 % were male. Common indications for EGD included epigastric pain, upper GI bleeding, dysphagia and dyspepsia; most children undergoing EGD required dilation of esophageal strictures. Indications for colonoscopy included hematochezia, chronic anemia, chronic diarrhea, chronic abdominal pain and evaluation for a primary source of adenocarcinoma. Of all endoscopies (upper and lower), 59.5% had diagnostic findings and the remainder (40.5%) was normal. EGD findings are shown in Table 1. Of the biopsies done on tumors and ulcers during EGD, 30 (2.2%) showed malignancy including adenocarcinoma in 22 (1.6%), squamous cell cancer in 3 (0.2%), and other malignancies in 5 (0.4%). Eight (0.6%) of the patients with malignancy were younger than 50 years of age, and 22(1.6%) were ? 50 years of age. The likelihood of finding malignancy was similar between males (1%) and females (1%). Eleven gastric ulcers were malignant (0.8%).

**Table 1:** Abnormalities diagnosed during EGD exams

FINDING	N (%)
Esophagitis	98(7.1)
Esophageal ulcer	7 (0.5)
Esophageal malignancy	3 (0.2)
Stricture	129 (9.4)
Varices	14 (1)
Foreign body	4 (0.2)
Gastritis	207 (15.09)
Gastric ulcer	108 (7.8)
Gastric malignancy	26 (1.9)
Duodenitis	38 (2.77)
Duodenal ulcer	177 (12.9)
Duodenal malignancy	1 (0.07)
Total positive	812 (59.3)
Normal	559 (40.7)
Total EGD	1371 (100)
EGD = esophagogastroduodenoscopy	

Interventions performed during EGD included biopsy in 73 (5.3%), stricture dilation in 129 (9.4%), esophageal variceal banding in 14 (1%), foreign body extraction in 4 (0.2%) and esophageal stent placement in 3 (0.2%). The commonest foreign bodies were meat, dentures and fish bones. Stents were placed under endoscopic control after dilation and endoscopic traversal of malignant strictures, and without fluoroscopy. After measurement of the length of the tumor and the distance from the incisors to the proximal and distal ends of the stricture, a guide wire was placed to the stomach. A mark indicating the correct distance was placed on the stent delivery catheter and the catheter was inserted over the guidewire until the mark was at the incisors. The stent was then deployed and the endoscope was reinserted to verify the exact position of the stent.

Colonoscopy findings are shown in Table 2. Cecal intubation rates per month of the study period from January to December 2015 were as follows (%): 30, 38, 49, 54, 66, 69, 73, 79, 82, 85, 87, 89. Of the malignancies, 21 (5.5%) were adenocarcinoma and 1 was a gastrointestinal stromal tumor (0.2%) and 8 (2.1%) were diagnosed in patients younger than 50 years of age. The likelihood of finding malignancy was similar between male (2.9%) and female (2.9%) patients. Therapeutic interventions performed during colonoscopy included 2 (0.3%) cases of rectal snare polypectomy. A comparison of our findings in 2015 and Dent’s findings in 1990 is shown in Table 3. Dent had a total of 1075 cases of upper GI endoscopy, with no colonoscopy reported. There were diagnostic findings in 620 (57%) cases in Dent’s study versus 59.5% in our study. Dent found a significantly greater percentage of pyloroduodenal ulcers, while we found significantly more gastric ulcers, esophagitis, esophageal strictures, and duodenitis.

**Table 2:** Abnormalities diagnosed during colonoscopy exams

Finding	N (%)
Hemorrhoid	83 (21.8)
Cancer	22 (5.8)
Colitis	64 (16.8)
Diverticulum	21 (5.5)
Benign polyp	26 (6.8)
Others	14 (3.7)
Total positive	230 (60.5)
Normal	150 (39.5)
Total colonoscopy	380 (100)

**Table 3:** Comparison of our EGD findings in 2015 with Dent’s findings in 1990

Finding	Dent 1990, N (%)	Kamdem 2015, N (%)	P value
Peptic ulcer disease (gastric or duodenal)	369 (34.3)	285 (20.7)	< 0.0001
Pyloroduodenal ulcer	353 (32.8)	177 (12.9)	< 0.0001
Gastritis	111 (10.3)	207 (15.09)	0.0005
Gastric cancer	37 (3.4)	26 (1.9)	0.0201
Esophagitis with or without hiatus hernia	27(2.5)	98 (7.1)	< 0.0001
Gastric ulcer	16 (1.5)	108 (7.8)	< 0.0001
Benign gastric polyp	9 (0.8)	-	0.0006
Esophageal varices	9 (0.8)	14 (1)	0.6792
Duodenitis	-	38(2.77)	< 0.0001
Esophageal stricture	-	129 (9.4)	< 0.0001
Miscellaneous	32 (3.1)	15 ( 1.09)	0.0009
Normal EGD	455 (42.3)	559 (40.7)	0.457
EGD = esophagogastroduodenoscopy			

Adverse events occurred in 3 of our cases including two cases of death during or soon after EGD. Both of these patients had active upper gastrointestinal bleeding and received pre-procedure resuscitation with intravenous normal saline, blood transfusion, and administration of supplemental oxygen. The first patient had active bleeding from a large gastric tumor and arrested suddenly during endoscopy, prior to any endoscopic treatment. The second patient had a large, actively bleeding duodenal bulb ulcer that was treated with injection of dilute epinephrine. Hemostasis was achieved, but the patient re-bled on the hospital ward and died during subsequent emergency surgery. The third adverse event occurred in a woman with multiple duodenal bulb ulcers who developed signs of acute abdomen in the ward following endoscopy exam. An abdominal X-ray showed free air under right diaphragm and she underwent emergent laparotomy with repair of a duodenal perforation and had a good post-operative recovery.

## Discussion

Mbingo Baptist Hospital (MBH), where this study took place, is a regional referral hospital located in Northwest Region of Cameroon, with both medical and surgical residency programs. Gastrointestinal endoscopy started in this facility in 2009, with 408 endoscopies performed that year, and there has been yearly growth since then. Endoscopy is performed by Cameroonian staff trained by local and experts who visit annually. Advantages of repetitive training in GI endoscopy have been demonstrated in previous studies [10-12]. During the study period an intensive care unit was opened at MBH and endoscopic measures used to treat hemorrhage included injection of 1:10,000 adrenaline and endoscopic band ligation. Major issues face endoscopists in subSaharan Africa, such as lack of equipment and the high cost of endoscopy equipment [13,14]. A complete examination was always accomplished during upper endoscopy unless an obstruction or patient non-cooperation was encountered. However colonoscopy was often incomplete, due to poor preparation, patient discomfort, presence of strictures or difficult anatomy, and skill of the endoscopist. Cecal intubation rates varied from about 30% at the beginning to about 90% by the end of the period of study, and improved in association with repetitive training of endoscopy staff by visiting endoscopists.

The commonest indication for EGD was epigastric pain. This finding is similar to other studies in Africa [5,15]. When compared to a 1990 report from Dent et al. regarding EGD in our region of Cameroon, we found peptic ulcers less often. The significant decrease in peptic ulcer may be due to the fact that studies in Cameroon as in other African countries have demonstrated the role of helicobacter pylori in causing gastritis and peptic ulcer [2,3,16,17] and empiric helicobacter pylori eradication treatment is commonly prescribed for treatment of dyspepsia at our hospital and in our region. However the availability of non-steroidal anti-inflammatory drugs (NSAIDS) has increased in our region during the past 25 years. NSAIDS and helicobacter pylori are independent risk factors for development of peptic ulcer disease [18] and NSAIDS might account for the higher prevalence of gastric ulcer in our series compared to Dent’s. We also found that esophagitis was significantly more common in 2015 compared to Dent’s experience in 1990. Gastroesophageal reflux disease (GERD) appears to be increasingly prevalent in many regions of the world, perhaps due to changes in diet, body mass index, physical activity, and smoking. Helicobacter pylori eradication may also increase the subsequent prevalence of GERD, by preventing helicobacter-associated atrophic gastritis with achlorhydria. Our findings of changing prevalence of peptic ulcer disease and esophagitis might also be due to other factors we could not assess, such as patient acceptance of endoscopy, cost of endoscopy, improvements in endoscopic technology, changes in referral patterns, and demographic changes.

We performed upper gastrointestinal endoscopy therapeutic procedures when indicated and feasible. The most common intervention performed during EGD was serial esophageal stricture dilations (129 endoscopic dilations on 13 patients). The most common cause of strictures was accidental caustic product ingestion by children. This caustic product is mainly used for artisanal production of soap. We performed esophageal variceal banding in 8 patients. We did not perform balloon dilation for gastric outlet obstruction as it was our practice to refer such patients for surgical intervention. Two patients died during or soon after EGD, both due to active gastrointestinal bleeding. We think this complication can be reduced in future with better selection and resuscitation of patients, availability of additional resuscitation equipment and assistance of an anesthetist during high risk procedures.

Our colonoscopy results are limited by frequent performance of incomplete colonoscopy at the start of the study period. The coecal intubation improved throughout the study period due to repetitive training. A retrospective study from Accra, Ghana reported a complete colonoscopy rate of 30%, while the recommended coecal intubation rate in the west is 90% [11,12].

## Conclusion

Digestive endoscopy is a useful diagnostic tool in our practice setting. The majority of patients undergoing EGD and colonoscopy had diagnostic findings. When compared to a published report from our region from 25 years ago, we found that pyloroduodenal ulcers were less common, but gastric ulcers and esophagitis were more common. These changes may be due to the advent of widespread helicobacter pylori eradication treatment, prevalence of HIV-related gastrointestinal disease and changes in diet, physical activity, NSAID use and smoking. Periodic on-site training can improve colonoscopy skills.

### What is known about this topic

- Gastrointestinal endoscopy is an appropriate diagnostic modality with a high diagnostic yield;
- Endoscopy therapy successfully treats some gastrointestinal conditions;
- Training in endoscopy improves skills and outcome.

### What this study adds

- Peptic ulcer disease is less common in Northwest Region Cameroon than 25 years ago - Esophagitis is more common, and digestive cancers are diagnosed at similar rates;
- Esophageal stricture and varices are the commonest conditions requiring endoscopic treatment in our setting;
- Periodic on-site mentorship is associated with improved colonoscopy cecal intubation rates.

## References

[1] Adam HA, Jonathan CO, Brian SA, Peter CO, Christopher WI (2014). Cotton and Williams’ Practical Gastrointestinal Endoscopy, The Fundamentals.

[2] Palmer DO, Watson KR, Allen MJ (1994). Helicobacter pylori Infection and peptic ulcer disease in Cameroon, West Africa. Journal of Clinical Gastroenterology.

[3] Ankouane Andoulo F, Noah Noah D, Tagni-Sartre M, Ndjitoyap Ndam EC, Ngu Blackett K (2013). Epidémiologie de l’infection à Helicobacter pylori à Yaoundé: de la particularité à l’énigme Africaine. The Pan African Medical Journal.

[4] Kuipers EJ (1999). Review article: exploring the link between Helicobacter pylori and gastric cancer. Aliment Pharmacol Ther.

[5] Misauno MA, Ismaila BO, Usman BD, Abdulwahab-Ahmed A, Achinge GI (2011). Spectrum of endoscopically diagnosed upper gastrointestinal diseases in Jos. Sahel Medical Journal.

[6] Dent AW (1990). Gastroscopy in a West African rural mission hospital. Tropical Doctor.

[7] Aduful HK, Naaeder SB, Darko R, Baako BN, Clegg-Lamptey JN, Nkrumah KN, Adu-Arye NA, Kyere M (2007). Upper gastrointestinal endoscopy at the Korle Bu Teaching Hospital Accra, Ghana. Ghana Med J.

[8] Dakubo JCB, Kumoji R, Naaeder SB, Clegg-Lamptey JN (2008). Endoscopic evaluation of the colorectum in patients presenting with haematochaezia in at Korle-Bu Teaching Hospital Accra. Ghana Med J.

[9] Dominguez RL, Crockett SD, Lund JL, Suazo LP, Heidt P, Martin C (2013). Gastric cancer incidence estimation in a resource-limited nation: use of endoscopy registry methodology. Cancer Causes Control.

[10] Ball JE, Osborne J, Jowett S, Pellen M, Welfare MR (2004). Quality improvement programme to achieve acceptable colonoscopy completion rates: prospective before and after study. BMJ.

[11] Bowles CJ, Leicester R, Romaya C, Swarbrick E, Williams CB, Epstein O (2004). A prospective study of colonoscopy practice in the UK today: are we adequately prepared for national colorectal screening tomorrow. Gut.

[12] De Jonge V, Sint Nicolaas J, Cahen DL, Moolenaar W, Ouwendijk RJ, Tang TJ, van Tilburg AJ, Kuipers EJ, van Leerdam ME (2012). Quality evaluation of colonoscopy reporting and colonoscopy performance in daily clinical practice. Gastrointest Endosc.

[13] Olokoba AB, Olokoba LB, Jimoh AA, Salawu FK, Ehalaiye BF (2008). Challenges of establishing an upper gastrointestinal tract endoscopy unit in a resource-poor setting. Res J Med Sci.

[14] Mandeville KL, Krabshius J, Ladep NG, Mulder CJ, Quigley EMM, Khan SA (2009). Gastroenterology in developing countries issues and advances. World J Gastroenterol.

[15] Aduful HK, Naaeder SB, Darko R, Baako BN, Clegg-Lamptey JN, Nkrumah KN (2007). Adu-276 Arye NA, Kyere M. Upper gastrointestinal endoscopy at the Korle Bu Teaching Hospital Accra, Ghana. Ghana Med J.

[16] Tanih NF, Dube C, Green E, Mkwetshana N, Clarke AM, Ndip LM (2009). An African perspective on Helicobacter pylori: prevalence of human infection, drug resistance, and alternative approaches to treatment. Ann Trop Med Parasitol.

[17] Fernando N, Holton J, Zulu I, Vaira D, Mwaba P, Kelly P (2001). Helicobacter pylori infection in an urban African population. J Clin Microbiol.

[18] Papatheodoridis GV, Sougioultzis S, Archimandritis AJ (2006). Effects of Helicobacter pylori and nonsteroidal anti-inflammatory drugs on peptic ulcer disease: a systematic review. Clin Gastroenterol Hepatol.

